# Limitation of super-resolution machine learning approach to precipitation downscaling

**DOI:** 10.1038/s41598-025-05880-7

**Published:** 2025-08-17

**Authors:** P. Jyoteeshkumar Reddy, Richard Matear, John Taylor, Marcus Thatcher

**Affiliations:** 1 Commonwealth Scientific and Industrial Research Organisation Environment, Hobart, TAS Australia; 2 Australian National University, Canberra, ACT Australia; 3 Commonwealth Scientific and Industrial Research Organisation Environment, Aspendale, VIC Australia

**Keywords:** Climate and Earth system modelling, Projection and prediction

## Abstract

The present study explores the potential of super-resolution machine learning (ML) models for precipitation downscaling from 100 to 12.5 km at hourly timescale using the Conformal Cubic Atmospheric Model (CCAM) data over the Australian domain. Two approaches were examined: the perfect approach, which trains the ML model using coarsened high-resolution data as input (i.e., CCAM 12.5 km data coarsened to 100 km), and the imperfect approach, which uses original coarse-resolution data as input (i.e., CCAM model simulation at 100 km resolution) and in both the cases high-resolution data (i.e., CCAM 12.5 km simulation) is used as target. In the perfect case, the ML model (ML_Perfect_) accurately reproduces high-resolution climatology and extremes. However, the ML_Perfect_ model with CCAM 100 km simulation data as input (i.e., in the imperfect setting) underestimates the magnitude of the output and introduces spatial inconsistencies, while the ML_Imperfect_ model captures high-resolution structures but underestimates extremes. This suggests that the super-resolution ML_Perfect_ model approach is inappropriate for precipitation downscaling because of the spatial inconsistencies between the coarse and high-resolution simulations. Additionally, we introduced sensitivity-based diagnostics beyond standard evaluation methods to understand model behaviour and identify structural issues. These diagnostics reveal that both models increase precipitation inputs non-linearly without creating spurious spatial relationships. However, the ML_Imperfect_ model outputs precipitation in high-altitude regions regardless of input, highlighting the structural issue of the ML_Imperfect_ model. Our study highlights the challenges in using super-resolution ML models for precipitation downscaling, introduces several useful diagnostics for assessing the super-resolution ML models and their physical realism, and provides ideas to explore to improve ML-based precipitation downscaling.

**Supplementary Information:**

The online version contains supplementary material available at 10.1038/s41598-025-05880-7.

## Introduction

General Circulation Models (GCMs) provide critical information on how our future climate will evolve under various greenhouse gas emission scenarios. These GCM climate projections typically have spatial grid resolutions of around 100 km in the atmosphere^1,2^ limiting their ability to accurately represent the local-to-regional scale atmospheric processes and accurately simulate the localized extreme events^3,4^. To enhance the spatial resolution of GCM projections, dynamical and statistical downscaling is applied. Dynamical downscaling is performed using the regional climate model (RCM). RCMs utilise the GCMs as lateral boundary conditions to simulate the physical processes at high spatial resolutions over a selected spatial domain. RCM’s high spatial resolution simulations are computationally expensive^5,6^ and this makes the dynamical downscaling of many GCM projections computationally challenging. However, downscaling using statistical and machine learning (ML) methods is computationally cheap and fast, enabling the downscaling of multiple ensembles of various GCMs^7–10^.

The potential utility of ML models for climate downscaling has spawned much interest, given their potential to dramatically reduce the computation cost and time required to generate high-resolution simulations. Several previous studies have applied ML methods like multilayer perceptron and support vector machines to downscale surface variables like temperature and precipitation^11,12^. Recently the advanced ML models based on the Convolutional Neural Networks (CNNs) are widely used for the precipitation downscaling following the super-resolution approach^12–18^. In precipitation downscaling, using the super-resolution approach ML models learns the mapping between the high-resolution precipitation and the coarse resolution precipitation data to generate the high-resolution precipitation. Super-resolution machine learning model training for precipitation downscaling is generally divided into two approaches: perfect downscaling and imperfect downscaling^9,19^. In the perfect downscaling approach, first the high-resolution precipitation is coarsened by conservative averaging or other averaging techniques to coarse resolution and then train the super-resolution ML model for downscaling the coarsened low-resolution precipitation to high-resolution target. Most studies have focused on the perfect downscaling approach for ML model training to perform precipitation downscaling^12,15,16,20,21^. They showed that the ML models perform well in downscaling the precipitation compared to the target high-resolution precipitation data when given with the perfectly aligned coarsened low-resolution precipitation as input. The perfect problem is useful because it provides a very clear way to demonstrate and assess the ability of ML models to learn the mapping between coarse and fine resolution and provide high-resolution information.

However, to realise the benefit of ML models in climate downscaling, one must solve the imperfect problem. In the imperfect downscaling problem, data from a low resolution model simulation is used to train the ML model for downscaling the low-resolution data to the high-resolution target data generated by a high-resolution simulation. In this case, the coarse-resolution simulation used as the input data is not perfectly aligned with the high-resolution simulation because the behaviour of the synoptic weather features is altered by the resolution of the model. For the imperfect problem, the ML models need to learn the biases in the coarse-resolution simulation as well as enhance its spatial resolution to emulate the high-resolution simulation. This is a much more challenging problem. However, the previous studies did not investigate the ML model capabilities for the imperfect problem^12,15,16,20,21^. In this study, we explore the potential of both the perfect and imperfect downscaling-based super-resolution ML models in downscaling precipitation over the Australian domain. We discuss the challenges of the imperfect problem and possible solutions. Linked to strategies to improve the solution of the imperfect problem is the need to further develop methods to assess the ML model.

The black-box nature, lack of physical understanding, and lack of robust evaluation of the complex ML models raise concerns about the applicability and the use of the downscaled data from the ML models. This requires further robust evaluation of the ML models in the downscaling context to help understand the behaviour of the ML model and the potential problems associated with the model. Explainable Artificial Intelligence (XAI) techniques are widely used to interpret and explain the behaviour of machine learning models in climate research^7,8,22–26^. Recent studies have utilized XAI methods, such as saliency maps, to assess machine learning models’ performance in downscaling climate variables, such as surface temperature^23^. The saliency maps technique quantifies the importance of inputs (for example, input variables and grid points) to a specific model prediction using gradient-based approximations. These gradient approximations map how inputs influence the ML output, but they don’t incorporate the complex non-linear processes in their explanations^22,26^ nor provide a concise and efficient way to assess the physical realism of the ML model. Here we propose a simple sensitivity-type method to understand the ML model behaviour in performing the super-resolution downscaling of precipitation. We evaluate the performance of super-resolution machine learning models using the new sensitivity-based diagnostics, which go beyond traditional validation frameworks. These diagnostics help us understand the model’s behaviour and uncover any structural issues that standard validation methods cannot detect.

## Results and discussion

**Fig. 1:**
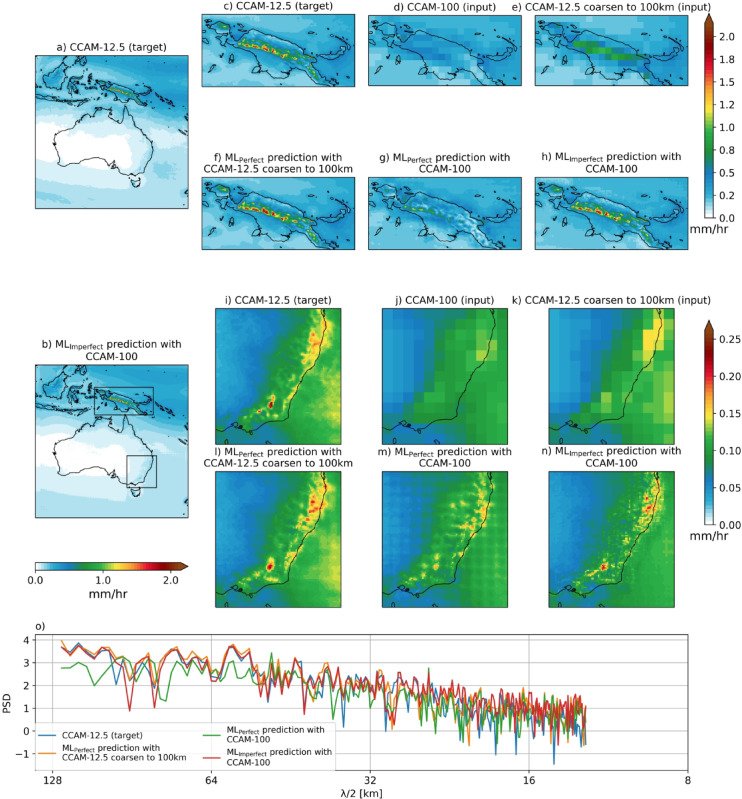
Climatology of hourly precipitation (mm/h) of CCAM 12.5 km target (**a**) and ML_Imperfect_ model predictions (**b**) over the study region during the test period. The top panels (**c**–**h**) show climatology over zoomed-in region of Papua New Guinea (PNG). Panel (**c**) shows CCAM-12.5 km target, (**d**) CCAM-100 km input, (**e**) CCAM-12.5 km coarsen to 100 km input, (f) ML_Perfect_ model prediction with 12.5 km coarsen 100 km input, (**g**) ML_Perfect_ model with CCAM 100 km input, and (h) ML_Imperfect_ model prediction climatology over the PNG region, respectively. Similar to top panel, middle panel (**i**–**n**) shows the climatology over the southeast Australia region (SEA). The line plot in the bottom panel (**o**) shows the power spectral density (shown only for mid-range and short wavelengths) of the climatology across the entire domain for different considered model predictions and the target. Maps are drawn using the Python Cartopy package (v0.24.1).

**Fig. 2:**
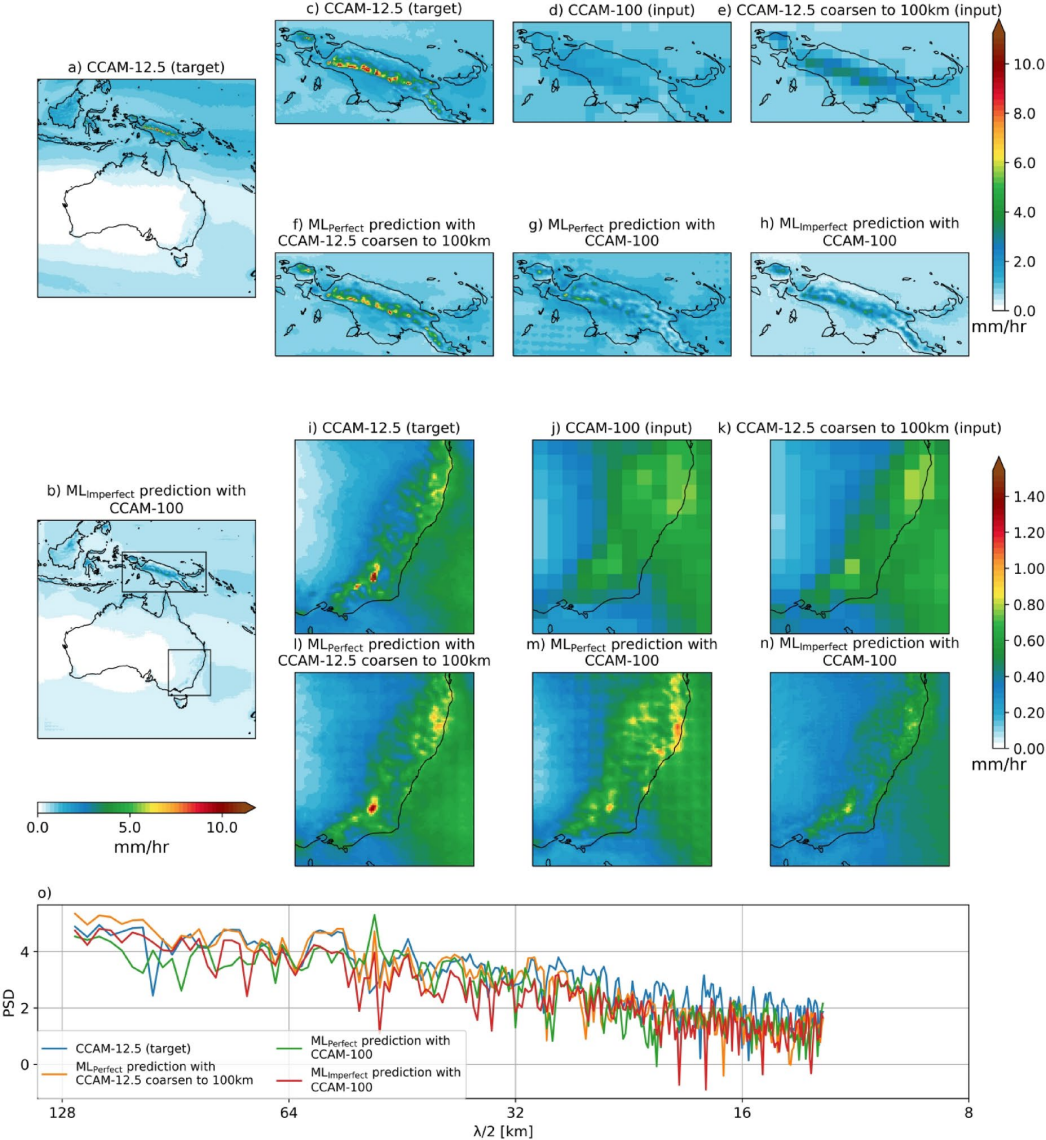
Same as Fig. 1 but for the 95^th^ percentile of the hourly precipitation during the test period. Maps are drawn using the Python Cartopy package (v0.24.1).

**Fig. 3:**
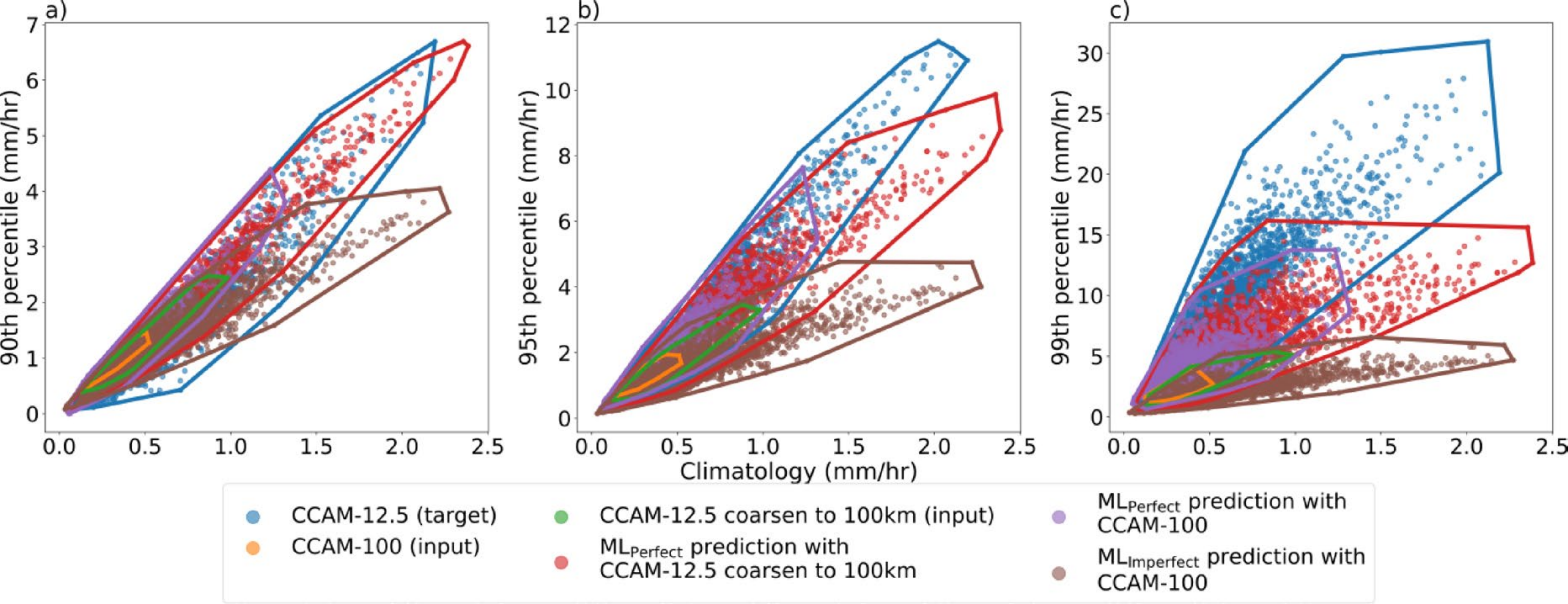
Scatter plot comparison of mean versus extreme relationship (climatology versus 90^th^ percentile (**a**), versus 95^th^ percentile (**b**), and versus 99^th^ percentile (**c**), respectively) among the CCAM-12.5 km target (blue), CCAM-100 km simulation (orange), CCAM-12.5 coarsen to 100 km input (green), ML_Perfect_ model predictions with CCAM-12.5 coarsen to 100 km input (red), ML_Perfect_ model with CCAM-100 km input (purple), and ML_Imperfect_ model predictions with CCAM-100 km input (brown).

For this study, we run CCAM at 12.5 km horizontal resolution (CCAM-12.5) and at 100 km horizontal resolution (CCAM-100). Both CCAM configurations are forced using spectral nudging for winds, air temperature and surface pressure from the fifth generation of the European Centre for Medium-Range Weather Forecasts atmospheric reanalysis (ERA5^27^) over the 1980 to 2020 period. The two CCAM simulations have similar large scale features that come from nudging to ERA5 but differ in how they represent synoptic weather. These two simulations produce significantly different rainfall, particularly in the regions with strong orography and high mean rainfall (Fig. 1). The large difference between the two simulations shows the challenge ML must overcome to downscale CCAM-100 to the resolution of CCAM-12.5. For example, the mean climatological hourly rainfall of CCAM-12.5 is about 5x the CCAM-100 values. The scaling increases further as one compares the extreme hourly rainfall with CCAM-12.5, 5x greater at 90^th^ percentile and 10x greater at the 99^th^ percentile than CCAM-100 (Figs. 2 and 3). The ML downscaling of hourly rainfall must correct the mean, add spatial variability and enhance extremes to emulate the behaviour of the CCAM-12.5 (Figs. 1 and 2). In the following discussion, we first demonstrate a super resolution ML model can accurately downscale hourly rainfall from the coarsen CCAM-12.5 at 100 km resolution to the CCAM-12.5 values (target data). We call this ML downscaling model, ML_Perfect_. We then apply the ML_Perfect_ to the CCAM-100 to see how well it can reproduce the CCAM-12.5 values. The downscaled rainfall lacks the variability present in CCAM-12.5 simulation, which motivates us to try another ML model. Finally, we train a super resolution ML to downscale the CCAM-100 values to CCAM12.5 and we call this downscaling model as ML_Imperfect_.

### Applicability of super-resolution ML approach to precipitation downscaling

First, we assess the ML models’ ability to predict the climatology of hourly precipitation for the test period (2012–2020), which we call the target data in Fig. 1a,c,i. The ML_Perfect_ with coarsen CCAM-12.5 input captures well the target data (Fig. 1a,b). The ML_Imperfect_ with CCAM-100 as input also captures well the climatological rain as shown in Papua New Guinea (PNG) and southeast Australia (SEA) (Fig. 1h,n). Both models represent the fine-scale spatial structure of the climatology well, even when zoomed in on complex orographic regions like PNG and SEA compared to the target. The ML_Perfect_ model better predicts the intensity of the fine-scale spatial pattern of the target data than the ML_Imperfect_ model (compare Fig. 1c–f,h,i–l,n). This is because ML_Perfect_ only needs to learn the mapping between the perfectly aligned input and target (Fig. 1c vs. e,i vs. k), and the coarsened input partly preserves the fine-scale spatial pattern compared to the low resolution CCAM-100 simulation (compare Fig. 1e–d,k–j). The ML_Imperfect_ has more difficult task because it must learn the mapping from input to the target, which includes learning the spatial inconsistencies (compare Fig. 1d,c).

For comparison, the CCAM-100 simulation was used as input to the ML_Perfect_ model (Fig. 1g,m). The resulting prediction fails to capture the spatial structure of climatology in the PNG and SEA regions (see Fig. 1g,m). Further, the prediction has high average rainfall over the southwestern parts of PNG instead in the central PNG region and creates a spatial mismatch of high average rainfall regions in the SEA domain as well compared to the target (compare Fig. 1c vs. g,i vs. m). This is because ML_Perfect_ seems to be learned the mapping between coarse input and target, which is sharpening and increasing the rainfall values with fine-scale spatial structure around the regions of moderate precipitation values, for example, the central PNG region (see Fig. 1e,f) and northeast parts of SEA domain (Fig. 1k,l). Hence, ML_Perfect_ with CCAM-100 simulation as input produces a high precipitation average in the southwest PNG region where the high rainfall climatology is seen in the CCAM-100 km simulation (Fig. 1d,g). This is also the same for the SEA domain; ML_Prefect_ sharpened and increased the rainfall average around the high average precipitation region of the CCAM-100 km simulation (Fig. 1j,m). The power spectral density (PSD; calculated using the 2-dimensional Fast Fourier Transform as mentioned in Reddy et al. (2023)) of climatology shows that the ML_Perfect_ with CCAM-100 km input underestimates the PSD at mid-range wavelengths compared to the target PSD (Fig. 1o). These results highlight the limitation of applying ML_Perfect_ to input from a coarse resolution CCAM simulation making ML_Perfect_ unsuitable for climate downscaling. In contrast, ML_Imperfect_ with the same low-resolution CCAM-100 input can reproduce the fine-scale spatial pattern of precipitation climatology of the target and appears to successfully downscale climatological rainfall (Fig. 1c vs. h).

Figure 2 shows the 95^th^ percentile maps of hourly precipitation of the CCAM 12.5 km target, for ML_Perfect_ with coarsen CCAM-12.5 to 100 km input, ML_Perfect_ with CCAM-100 input and ML_Imperfect_ with CCAM-100 input. Similar to the climatology results, ML_Perfect_ with coarsen CCAM-12.5 input reproduces the fine-scale spatial pattern of extremes (95^th^ percentile) well in the complex orographic regions of PNG (Fig. 2c,f) and SEA (Fig. 2i,l). However, ML_Perfect_ with CCAM-100 input is not able to get the fine-scale spatial pattern of extremes and has spatial inconsistencies similar to the climatology as mentioned above (Fig. 2g vs. c,m vs. i). Whereas ML_Imperfect_ can reproduce the fine-scale spatial structure of extremes but underestimates the magnitude (Fig. 2h vs. c,n vs. i).

We evaluate the ML performance by examining the relationship between climatology and extremes (90^th^, 95^th^, and 99^th^ percentiles) by plotting the climatological mean value versus the corresponding extreme value (Fig. 3). The relationship allows one to assess the ML models without focusing on an exact grid point comparison, which is flawed because of chaotic weather processes. A comparison of the CCAM-12.5 simulated relationship to the CCAM-100 simulation shows the low resolution substantially underestimates both the climatological extreme rainfall. CCAM-100 is a poor reflection of the CCAM-12.5. ML_Perfect_ with CCAM-12.5 coarsen input reproduces the mean and extreme relationship of the target for the 90^th^ and 95^th^ percentiles but underpredicts the 99^th^ percentile values. For the 99^th^ percentile, ML_Perfect_ underestimates the relationship between the mean rainfall and the extreme value with an underprediction of the extreme values. ML_Perfect_ with CCAM-100 input poorly predicts the mean and extreme relationship by underestimating both the mean and extreme values. ML_Imperfect_ with CCAM-100 input slightly underestimates the extreme values in the mean versus extreme relationship at the 90^th^ percentile compared to the target (Fig. 3a). The ML_Imperfect_ under prediction of the extremes become more evident for rainfall extremes above the 90^th^ percentile with an underprediction by 2.5x and 6x for the 95^th^ and 99^th^ percentile, respectively (Fig. 3b,c).

### ML model sensitivity investigation – explainable ML experiments

**Fig. 4:**
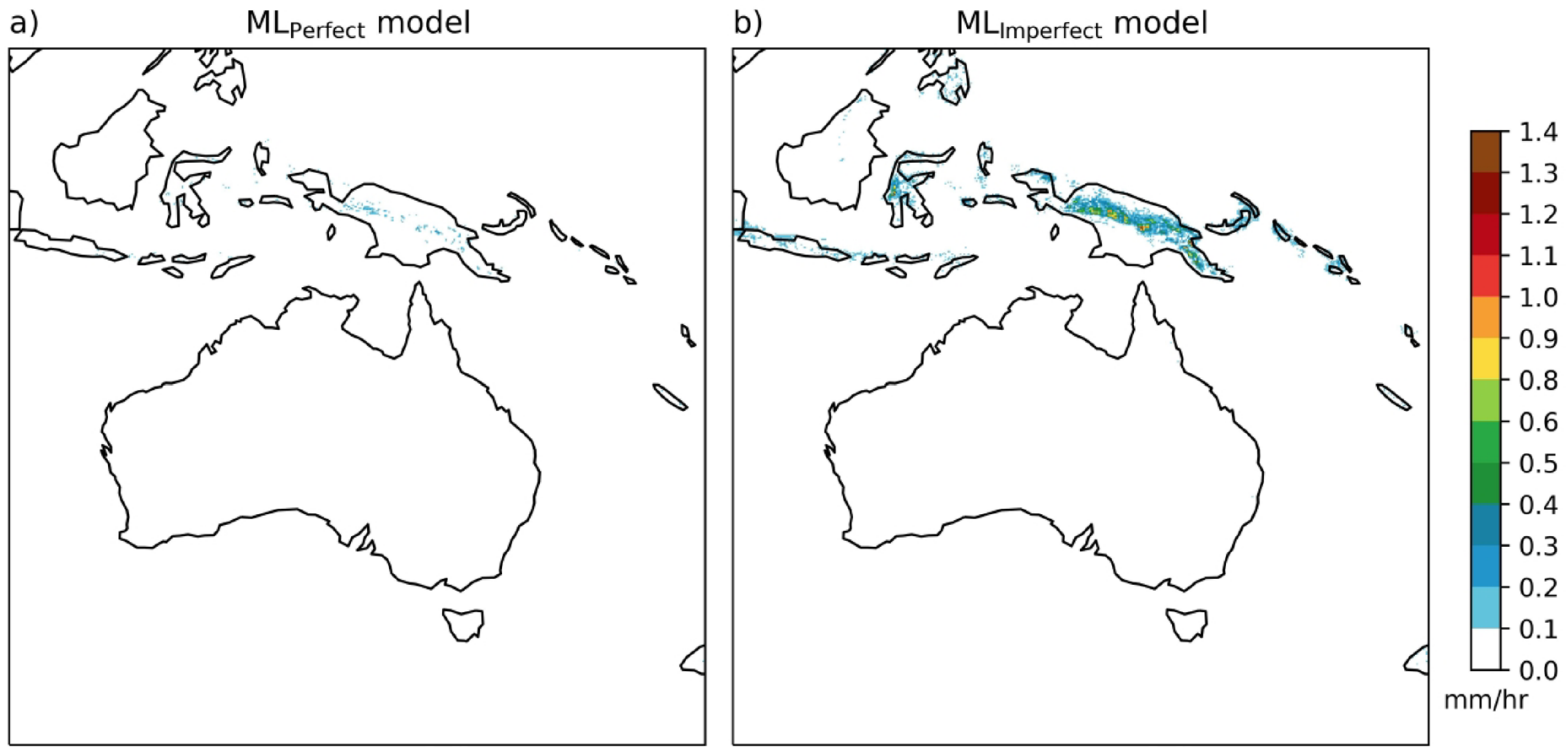
ML_Perfect_ (**a**) and ML_Imperfect_ (**b**) model output precipitation (at 12.5 km) when provided zero coarse precipitation input (at 100 km) on all grid points and the additional orography input unchanged. Maps are drawn using the Python Cartopy package (v0.24.1).

**Fig. 5:**
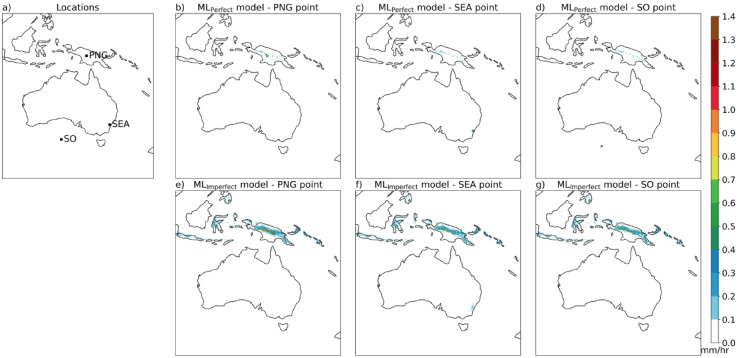
ML_Perfect_ and ML_Imperfect_ model output precipitation (at 12.5 km) when provided zero coarse precipitation input (at 100 km) on all grid points except a small precipitation perturbation of 0.5 mm/h at the three selected points (one in Papua New Gunia (PNG), second in South East Australia (SEA), and the third in Southern Ocean (SO) as shown in (**a**)), each point perturbation at a time and the orography input is unchanged. ML_Perfect_ and ML_Imperfect_ model output precipitation when perturbed at the PNG point with orography unchanged are shown in (**b**) and (**e**), respectively. The ML_Perfect_ and ML_Imperfect_ model outputs when perturbed at SEA and SO points are shown in (**c**), (**f**) and (**d**), (**g**), respectively. Maps are drawn using the Python Cartopy package (v0.24.1).

To understand the behaviour of the ML model for precipitation downscaling we performed several input perturbation sensitivity experiments. First, we present the results of the ML_Perfect_ and ML_Imperfect_ predictions when the input is zero precipitation values at all grid points with the additional orography input (shown in Fig. 4). The ML_Perfect_ model predicts zero precipitation everywhere except in PNG high altitude regions with very small values < 0.2 mm/h (Fig. 4a). However, ML_Imperfect_ with zero rainfall input predicts zero precipitation in the non-tropical regions of the domain and precipitation values of around 0.2–0.9 mm/h in the high-altitude tropical regions of the domain, particularly the elevated parts of the PNG region (Fig. 4b). The ML_Imperfect_ rainfall prediction is converting the orographic input into precipitation outputs (accounts up to 40% of the mean value). To further understand the effects of the orography signal on the ML-predicted rainfall, we conducted the input perturbation experiments at the three selected locations in the domain. Three locations are chosen in the high-altitude PNG region, SEA land region, and the Southern Ocean (SO) region, respectively (see Fig. 5a). Now, the input perturbation with 0.5 mm/h is performed at these locations, with the rest of the grid points are zeros. With this point perturbation at all three locations, the ML_Perfect_ predictions are concentrated at and near the perturbation point with very small precipitation (< 0.1 mm/h) in the high-altitude PNG regions, which is negligible (Fig. 5b–d). However, ML_Imperfect_ predicts precipitation around 0.5–0.9 mm/h over the high-altitude tropical regions of the domain irrespective of perturbations at the three selected locations (Fig. 5e–g). Further, we performed the input perturbations with 1 mm/h at three selected locations and the results are similar to the 0.5 mm/h input perturbation (Fig. S3). ML_Imperfect_ predicts precipitation at the high-altitude regions of the domain no matter the input, because it is getting the signal from the additional orography input. ML_Imperfect_ is utilising the topography in the model to map CCAM-100 to the CCAM-12.5 simulation by adding rainfall where it consistently underestimates rainfall resolution enhancements.

**Fig. 6:**
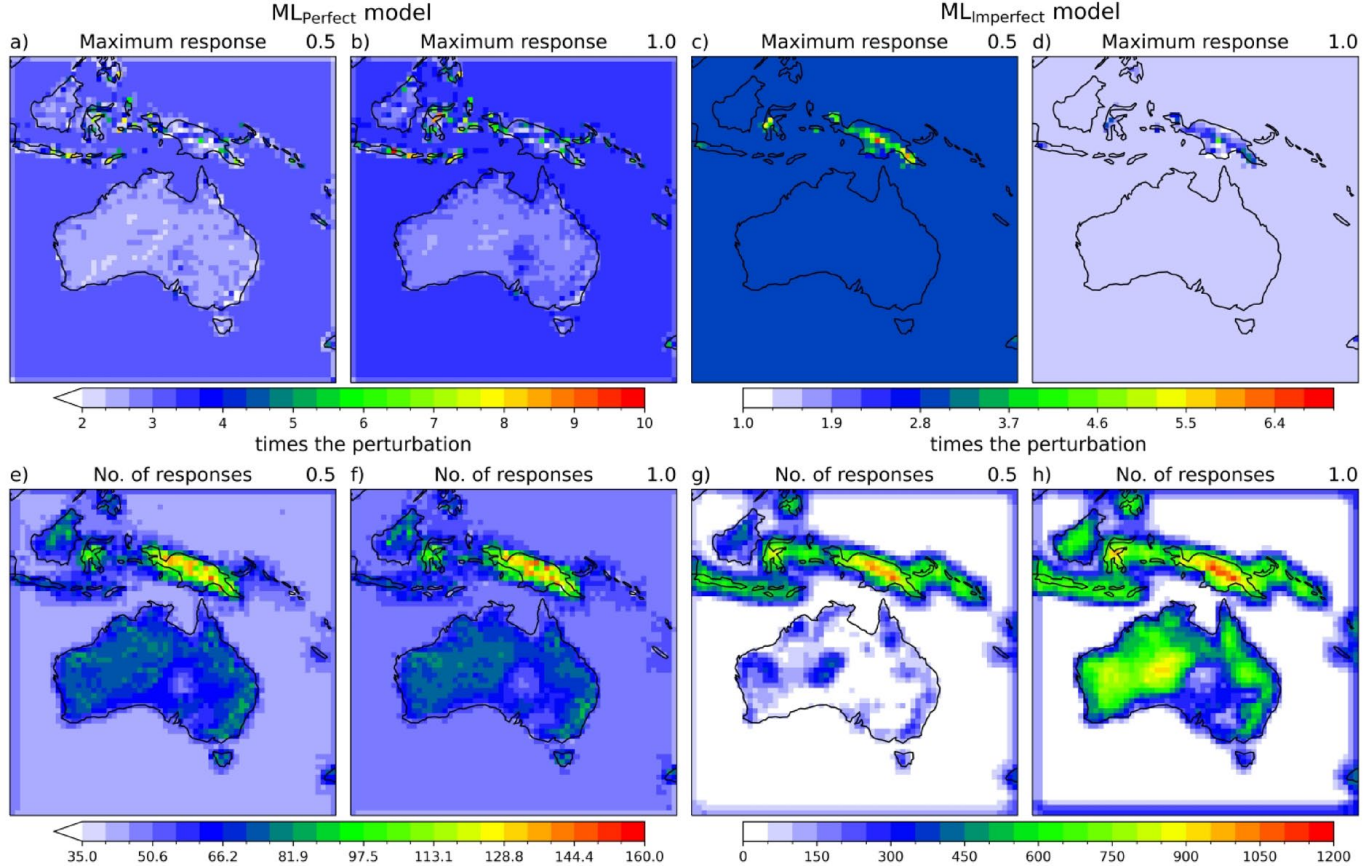
ML_Perfect_ model response diagnostics (maximum response (**a**) and number of responses (**e**)) when perturbed with 0.5 mm/h input at a particular grid point and made rest all grid points as zero; and in the same way iteratively executed at all grid points. ML_Perfect_ model response diagnostics when perturbed with 1 mm/h input are shown in subplots (**b**; maximum response) and (**f**; number of responses). ML_Imperfect_ model response diagnostics when perturbed with 0.5 and 1 mm/h input are shown in subplots (**c**; maximum response), (**g**; number of responses) and (**d**; maximum response), (**h**; number of responses), respectively. For more details about model response diagnostics refer to data and methods section. Maps are drawn using the Python Cartopy package (v0.24.1).

Here, we present the results of ML prediction for various input perturbations to understand and explain the behaviour of super-resolution ML models in precipitation downscaling. Figure 6 shows the model response diagnostics (maximum response and number of responses) of ML_Perfect_ and ML_Imperfect_ when perturbed with 0.5 and 1 mm/h. ML_Perfect_ model amplified the input perturbation across all the regions of the domain, i.e., when perturbed with 0.5 mm/h the maximum response ranges from around 2–7.5 times the perturbation and with 1 mm/h the maximum response ranges from 2 to 10 times the perturbation (Fig. 6a,b). Further tested with perturbations 5 and 10 mm/h, which resulted in maximum response ranges around 2.38–5 and 3–5 times the perturbation, respectively (Fig. S4a,b). The ML_Perfect_ shows a non-linear response with the scaling factor changing with the magnitude of the input perturbations. Consistent with sharpening up the coarse rainfall input, the maximum response is greater than 1. Given the 8x downscaling in the latitude and longitude, if the input rain was concentrated in just one grid cell of the fine resolution grid, the maximum response would be 64x the input value. The maximum response of the ML_Perfect_ varies across the domain such as some parts of the tropical land regions have the highest maximum response values, and some parts of the orographic regions over Australia have the lowest maximum response values compared to the other regions of the domain. This is because in the orographic regions the ML_Perfect_ model responded at many grid points, which probably means that the model is spreading out the input precipitation over neighbouring grid points with not spiking it too much at or next to the perturbed point (Fig. 6e,f).

Further, we have looked at the impact distance, to see how far the model response extends from the input perturbation. Results show that the maximum value of impact distance at all grid points is around 500 km (not shown), which is very close to the input perturbation grid point. This suggests that the ML_Perfect_ model did not learn any spatially spurious relationships. This is because the ML models are fully convolutional, where the filters/kernels mostly learn the spatially constrained information over a finite space. Similar to the ML_Perfect_ model, ML_Imperfect_ model extrapolated the input perturbations across the domain with maximum values over the PNG region. Perturbing with 0.5 mm/h the ML_Imperfect_ model responses range from around 2–6 times the perturbation and with 1 mm/h perturbation the model responses range from around 1.3–3.4 times the perturbation. The ML_Imperfect_ model responses are not varying much over the domain except in the PNG region, where the large variations are seen (Fig. 6c,d). ML_Imperfect_ fails to produce much scaling up of the input for the rest of the domain. ML_Imperfect_ does much less amplification of the input rainfall than the ML_Perfect_. There is strong non-linearity in the number of maximum responses in the ML_Imperfect_ in Australia. Doubling the input rainfall leads to more than 4x more responses. Consistent with the reduced amplification of the rainfall input, the ML_Imperfect_ spread the input rainfall of 1 mm/h over an order of magnitude more fine-resolution grid points than ML_Perfect_. The ML_Perfect_ and ML_Imperfect_ behave very differently in both the maximum response and in the number of responses and how it changes in input value - one responding to the bias and one correcting missing features.

## Conclusions

We explored the potential of super-resolution machine learning (ML) model approaches to downscale the precipitation from 100 km to 12.5 km spatial resolution over the Australian domain. For this, we investigated two super-resolution ML model training approaches for precipitation downscaling named perfect and imperfect. In the perfect downscaling case ML_Prefect_ gets the coarsened CCAM-12.5 simulated precipitation to 100 km resolution as input and is trained to predict the CCAM-12.5 precipitation. In the perfect case, ML_Prefect_ learns the downscaling function between the perfectly aligned coarsened low-resolution input and high-resolution target. In the imperfect downscaling case, ML_Imperfect_ gets low-resolution CCAM-100 km simulated rainfall as input and is trained to predict the CCAM-12.5 simulated rainfall. This is the climate downscaling using ML. ML_Perfect_ model with coarsened CCAM-12.5 input performed well in reproducing both the climatology and extremes (i.e., 95^th^ percentile). However, if ML_Perfect_ is given CCAM-100 simulated rainfall as input, the resulting prediction underestimates the climatological and extreme rainfall with spatial inconsistencies with the CCAM-12.5 rainfall. This is because the ML_Perfect_ focused on learning only downscaling and did not learn to correct the systematic differences in rainfall between CCAM-100 and CCAM-12.5. This shows the ML_Perfect_ is not applicable for climate downscaling the precipitation from a coarse resolution simulation to a finer resolution. In contrast, ML_Imperfect_ using CCAM-100 rainfall input to predict CCAM-12.5 rainfall output reproduced the climatological rainfall magnitude and spatial features of extremes. However, ML_Imperfect_ underestimated the magnitude of the extremes.

We introduced the sensitivity-based diagnostics to elucidate the ML behaviour and to identify potential issues. The sensitivity analysis showed the ML_Perfect_ and ML_Imperfect_ behaved differently. For the ML_Perfect_, there was a non-linearly increase in the response with increasing value of the inputs with the response being concentrated near the input point. In the high rainfall regions of the tropics, the rainfall scaling of the input is very patchy, consistent with the fine-scale features in the climatological and extreme rainfall of the region. The limited extent of the ML_Perfect_ response (i.e., the number of responses) is consistent with an ML model that is trying to downscale and enhance fine features of the rainfall. For ML_Imperfect_ there is also a non-linearly increased rainfall with an increase in the input rainfall. However, ML_Imperfect_ has an obvious response related to the orography of the tropics, which is associated with background rainfall. This unphysical behaviour is revealed from the new diagnostics used in this study, which would not have been identified using the standard evaluation methods such as looking at global grid point-based metrics like mean square error and peak signal-to-noise ratio. The feature of the ML_Imperfect_ sensitivity analysis is the much spatially extensive to a single input of rainfall with the tropics and western and northeastern Australia having an order magnitude number of responses than ML_Perfect_. ML_Imperfect_ is distributing the input of a single grid point rainfall over much larger regions. Our sensitivity experiments show the ML_Imperfect_ has unphysical behaviour in high altitude regions and needs further refinements before it can be used for climate downscaling of rainfall.

Bias correction methods such as quantile mapping (QM)^28^ and semi-parametric quantile mapping (SPQM)^29^ to name a few, which perform better in correcting the biases, can be applied particularly to the ML_Imperfect_ model, which underestimates extremes. However, our results suggest that the ML_Imperfect_ model has a structural issue of outputting precipitation in high-altitude regions regardless of input. However, precipitation downscaling with machine learning models is highly region-specific, as mentioned in the previous section. Hence, the ML_Imperfect_ model output in the less complex orographic regions to plains is useful, and applying the bias correction methods to these regions could improve the representation of precipitation output, particularly the extremes, which is outside the scope of the present study. Further, to improve the representation of extremes, modifications to the loss function, such as exponential and generative adversarial network (GAN) based adversarial losses, could be applied in future studies^30–32^. Furthermore, to address the uncertainties with the ML_Imperfect_ model, generative ML modelling approaches like GANs and diffusion models, to name a few, could be explored in future studies^32^. This work has some limitations, particularly due to biases inherent in using CCAM output for training. Since the ML models are trained on this dynamically downscaled data, any existing biases are carried over, meaning the model’s performance and evaluation is restricted by the quality of the training data. Nevertheless, interrogating the ML model is crucial to building confidence in the model that it is not producing unphysical behaviour. The input diagnostic present here provide a powerful way to investigate ML model behaviour, and they can be easily deployed for assessing the super-resolution downscaling ML models. For more sophisticated ML models they may require further development but the idea of using different inputs to the ML model to assess its predictions is a powerful way to elucidate the ML model behaviour and assess its realism and should become a standard way of assessing the ML models.

## Data and methods

### Data

In this study, we use the Conformal Cubic Atmospheric Model (CCAM^33,34^). This is a variable resolution global climate model based on a cubic grid, where the grid can be focused over a region without introducing lateral boundaries. Hourly precipitation data from 1980 to 2020 was provided at a coarse resolution (at 100 km for the globe using a C96 grid (CCAM-100)) and at a high-resolution (at 12.5 km, focused over Australasia with a C384 grid (CCAM-12.5))^33,34^. The fifth generation of the European Centre for Medium-Range Weather Forecasts (ECMWF) atmospheric reanalysis (ERA5 ^27^) data is used to drive the CCAM using spectral nudging for winds, air temperature and surface pressure. We selected the ML model training data from 1980 to 2012 (until 15th September 2012; approximately 286720 samples) and test data from 2012 (from 15th September 2012) to 2020 (i.e., approximately 72704 samples) based on the commonly applied 80−20 split rule. Before the model training in the data preprocessing step, the precipitation and static orography data used in model training are normalized based on the min-max normalization using the training period’s minimum and maximum values. Following the previous study^10^ the model input normalized data is then scaled to 100 because most of the hourly precipitation data has small values, and scaling them to 0–1 produces nearly zero values.

### Super-resolution machine learning based downscaling methods

#### Model architecture

We develop a super-resolution ML model using the deconvolution layers with stepwise static orography input called super-resolution deconvolution network – stepwise orography (SRDN-SO)^10^. SRDN-SO model takes in 100 km of precipitation data input and outputs the 12.5 km high-resolution precipitation, i.e., 8× resolution enhancement. SRDN-SO model has an input, three main hidden deconvolution layers, and three convolution layers at the end. Each deconvolution layer consists of 64 filters of 7 × 7 filter size and is activated non-linearly with rectified linear unit (ReLU). Each deconvolution layer performs a 2× resolution enhancement. The first convolution layer with a filter of 1 × 1 filter size linearly maps the last deconvolution layer feature maps to the next convolution layer. Next follows the second convolution layer with 64 filters of size 7 × 7 with ReLU activation. The last convolution layer with a filter of 1 × 1 size linearly maps the second convolution layer feature maps to the output. The static orography is provided at multiple steps as the feature maps, i.e., 50 km and 25 km orography data, are appended as a feature map to the first and second deconvolution layer’s feature maps, respectively, and 12.5 km orography data is concatenated with the first convolution layer output. To avoid overfitting, L2 regularization with a 1e−9 regularization factor is applied for all the convolution and deconvolution layers in the network. SRDN-SO model architecture is shown clearly in Fig. S1.

#### Model training

Super-resolution ML model training for downscaling is broadly categorized into perfect and imperfect downscaling cases^9,19^.

#### Perfect downscaling approach

In the perfect downscaling approach, the ML model is trained with a high-resolution target and the coarse-resolution model input, which is the upscaled version of the same high-resolution target. We call this model ML_Perfect_ to reflect it was trained for the perfect case, where the CCAM high-resolution precipitation data (12.5 km resolution) is conservatively interpolated to coarse resolution (100 km)^10^. In this case, the input and target data are perfectly aligned, with both having the same weather features, simplifying the model training. The ML_Perfect_ only needs to learn the spatial mapping of the coarse resolution spatial information to fine-scale resolution^9,19^.

ML_Perfect_ is trained with mean squared error (MSE) as a loss function. Adam optimizer with a learning rate of 3 × 10^− 3^ and a learning rate reduction factor of 0.1 is applied when the model does not improve the loss value for ten epochs with a minimum learning rate of 1 × 10^− 5^ is used for model training. The model loss value converges at the 30th epoch; further training doesn’t improve the loss (not shown). The model loss curve represents a good fit without overfitting the data (see Fig. S2). We have chosen the model hyperparameters based on the minimum validation loss and trained the model until the loss curve converges. To test ML_Perfect_, we evaluated its predictions using a coarse version of the high-resolution CCAM-12.5 simulation as input and with CCAM-100 simulation as input.

#### Imperfect downscaling approach

In the imperfect downscaling case, the ML model is trained using the high-resolution target data (CCAM-12.5) and input from the coarse resolution 100 km simulation (CCAM-100). This trained model, we call ML_Imperfect_, must learn the spatial inconsistencies between the CCAM 100 km and the CCAM 12.5 km data. This requires ML_Imperfect_ to learn the coarse to fine scale spatial relationships and the larger scale model differences of the coarse and fine scale model. Similar to ML_Perfect_ model, ML_Imperfect_ is also trained with MSE as a loss function for 60 epochs because the model loss converges at the 60th epoch. The ML_Imperfect_ model loss curve shows a good fit without any sign of overfitting (Fig. S2). ML_Imperfect_ is evaluated against the target CCAM-12.5 simulation with CCAM 100 km simulation as input.

### A simple explainability and evaluation method for machine learning based super-resolution downscaling

Here we propose a simple sensitivity-type method to understand the ML model behaviour in performing the super-resolution downscaling of precipitation. In this method, first, we perturb a coarse input grid point with a precipitation value of 0.5 mm/h, with the rest of the grid points made to zero; with this input, we then record the model-predicted responses. This is iteratively performed across all the coarse grid points. The model responses less than 0.1 mm/h are considered as noise and are removed. Further, we input all zeros at all coarse input grid points and record the model output, and this is used as an additional threshold for recording the model output responses for the input perturbation. The model responses for perturbed inputs less than the all zeros-input based model response value at a particular grid point are considered noise and removed. From these responses, we compute two key diagnostics to assess the model behaviour: one is the maximum response, which is the maximum model predicted value, and the second is the number of responses, which is the number of grid points with values greater than 0.1 mm/h in the model predictions. Maximum response and number of responses represent how rainfall at the coarse grid is mapped to the fine resolution grid. Further, we considered the impact distance, which is the maximum distance between the input perturbed grid point to the farthest grid point greater than 0.1 mm/h in the model predictions. Impact distance represents the spatial region of influence, i.e., how far the model response is from the input perturbation. Finally, to assess the non-linearity and extrapolation capabilities of the ML model, we perform the same sensitivity experiments with input perturbation values of 1, 2, 5, and 10 mm/h.

## Supplementary Information

Supplementary Information 1.

## Data Availability

ERA5 reanalysis data used to drive the CCAM model is freely available at: 10.24381/cds.bd0915c6. The coarse and high-resolution CCAM data can be made available upon request to the corresponding author.
