# Supplementary material for: Limitation of super-resolution machine learning approach to precipitation downscaling
Source: Sci Rep. 2025 Aug 17;15:30070. doi: 10.1038/s41598-025-05880-7 (PMC12358561; doi:10.1038/s41598-025-05880-7)
Supplement: Supplementary file 1 — Supplementary Information 1. [file 41598_2025_5880_MOESM1_ESM.pdf]

Limitation of super-resolution machine learning approach to precipitation downscaling

*P. Jyoteeshkumar Reddy[1, *], Richard Matear[1], John Taylor[2], Marcus Thatcher[3]*

[1] Commonwealth Scientific and Industrial Research Organisation Environment, Hobart, TAS, Australia

[2] Australian National University, Canberra, ACT, Australia

[3] Commonwealth Scientific and Industrial Research Organisation Environment, Aspendale, VIC, Australia

[*] Corresponding author: P. Jyoteeshkumar Reddy (jyoteesh.papari@csiro.au)

Supplementary material

Contents

Figures S1-S4

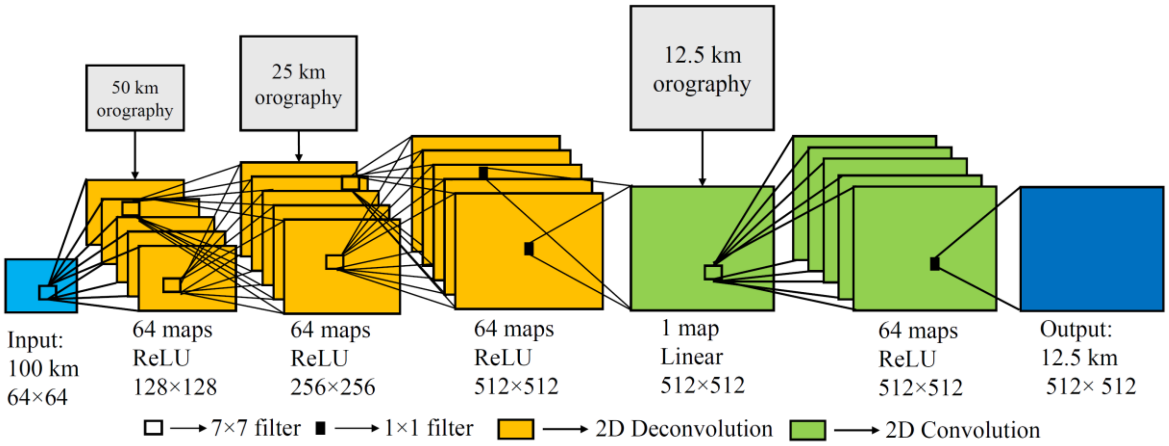

Fig. S1 An overview of the SRDN-SO model architecture.

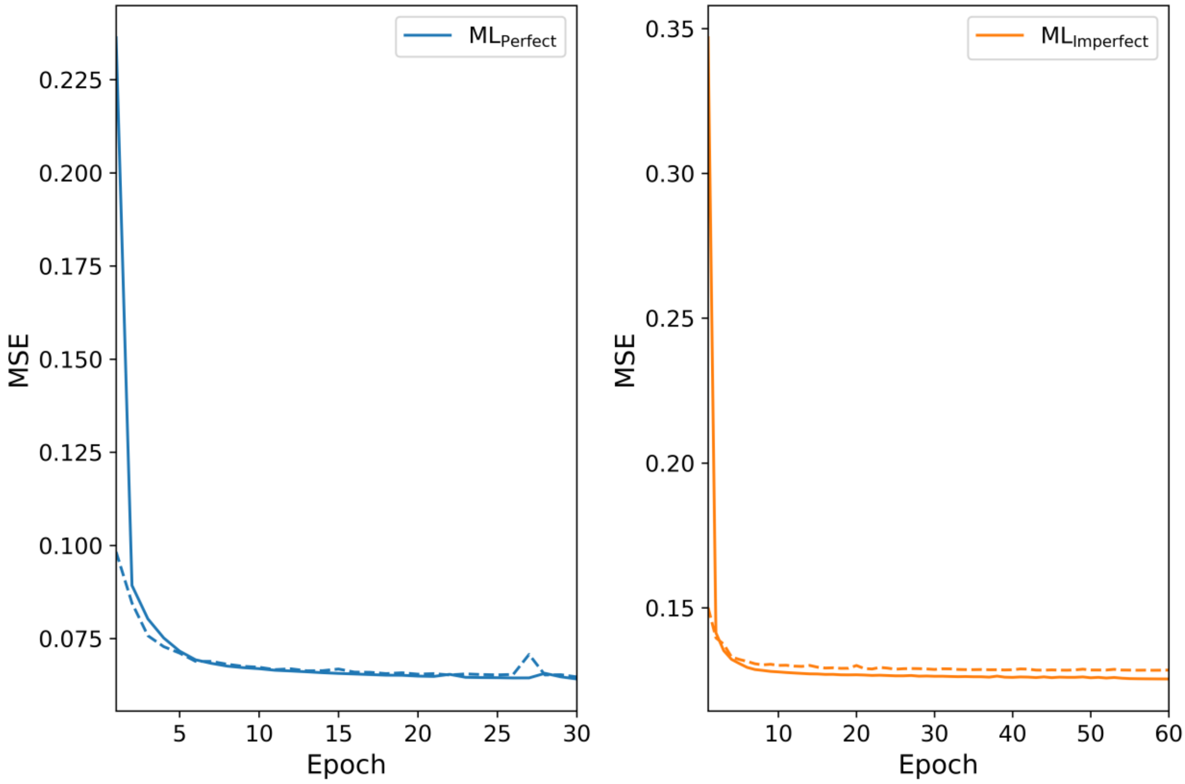

Fig. S2 Loss (MSE: Mean squared error) curve of the $ML_{Perfect}$ (left) and $ML_{Imperfect}$ (right) models. Solid line represents train data loss and dashed line shows test data loss.

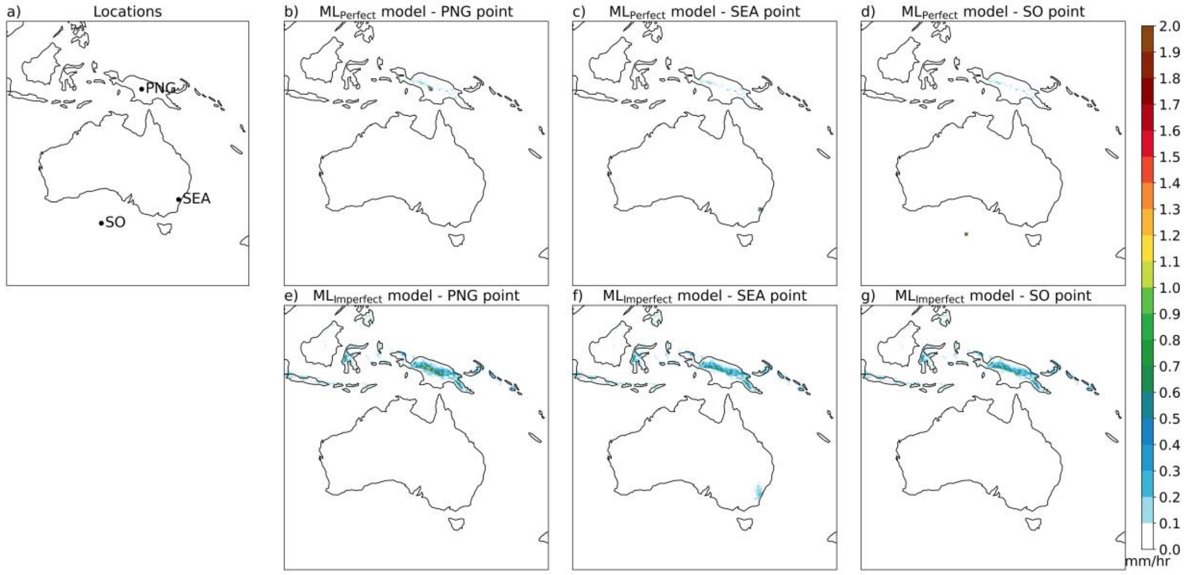

Fig. S3 $ML_{Perfect}$ and $ML_{Imperfect}$ model output precipitation (at 12.5 km) when provided zero coarse precipitation input (at 100 km) on all grid points except a small precipitation perturbation of 1.0 mm/hr at the three selected points (one in Papua New Gunia (PNG), second in South East Australia (SEA), and the third in Southern Ocean (SO) as shown in (a)), each point perturbation at a time and the orography input is unchanged. $ML_{Perfect}$ and $ML_{Imperfect}$ model output precipitation when perturbed at the PNG point with orography unchanged are shown in (b) and (e), respectively. The $ML_{Perfect}$ and

$ML_{Imperfect}$ model outputs when perturbed at SEA and SO points are shown in (c), (f) and (d), (g), respectively. Maps are drawn using the Python Cartopy package (v0.24.1).

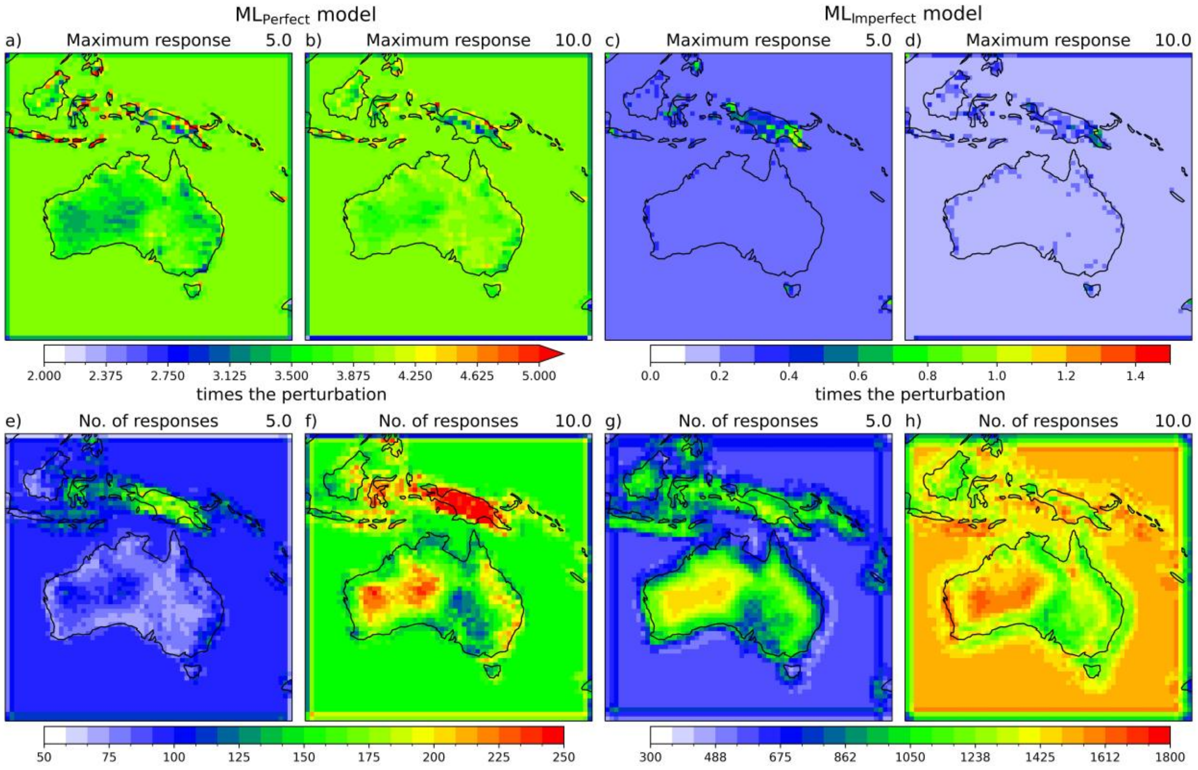

Fig. S4 $ML_{Perfect}$ model response diagnostics (maximum response (a) and number of responses (e)) when perturbed with 5 mm/hr input at a particular grid point and made rest all grid points as zero; and in the same way iteratively executed at all grid points. $ML_{Perfect}$ model response diagnostics when perturbed with 10 mm/hr input are shown in subplots (b; maximum response) and (f; number of responses). $ML_{Imperfect}$ model response diagnostics when perturbed with 5 and 10 mm/hr input are shown in subplots (c; maximum response), (g; number of responses) and (d; maximum response), (h; number of responses), respectively. For more details about model response diagnostics refer to section 2.3. Maps are drawn using the Python Cartopy package (v0.24.1).
